# Occupational, physical, sexual and mental health and violence among migrant and trafficked commercial fishers and seafarers from the Greater Mekong Subregion (GMS): systematic review

**DOI:** 10.1186/s41256-018-0083-x

**Published:** 2018-10-01

**Authors:** Nicola S. Pocock, Long Hoang Nguyen, Don Eliseo Lucero-Prisno III, Cathy Zimmerman, Siân Oram

**Affiliations:** 1 Department of Global Health and Development, Faculty of Public Health and Policy, 15-17 Tavistock Place, London, WC1H 9SH UK; 2 United Nations University International Institute of Global Health, UKM Medical Centre, Jalan Yaacob Latif, Bandar Tun Razak, 56000 Cheras, Kuala Lumpur Malaysia; 3 School of Medicine and Pharmacy, Vietnam National University, No. 144 Xuan Thuy Street, Cau Giay district, Hanoi, Vietnam; 4 Department of Public Health, Emerging and Interdisciplinary Sciences Building (ES) South Campus, Xi′an Jiaotong-Liverpool University, 111 Ren′ai Road, Suzhou Dushu Lake Science and Education Innovation District, Suzhou Industrial Park, Suzhou, 215123 People’s Republic of China; 5 Section of Women’s Mental Health, Institute of Psychiatry, Psychology and Neuroscience, 16 De Crespigny Park, Kings College London, London, SE5 8AF UK

**Keywords:** Migrant fishermen, Human trafficking, Seafarers, Migrant workers

## Abstract

**Background:**

Little is known about the health of GMS commercial fishers and seafarers, many of whom are migrants and some trafficked. This systematic review summarizes evidence on occupational, physical, sexual and mental health and violence among GMS commercial fishers/seafarers.

**Methods:**

We searched 5 electronic databases and purposively searched grey literature. Quantitative or qualitative studies reporting prevalence or risk of relevant outcomes were included. Two reviewers independently screened articles. Data were extracted on nationality and long/short-haul fishing where available.

**Results:**

We identified 33 eligible papers from 27 studies. Trafficked fishers/seafarers were included in *n*=12/13 grey literature and *n*=1/20 peer-reviewed papers. Among peer-reviewed papers: 11 focused on HIV/AIDS/sexual health; nine on occupational/physical health; one study included mental health of trafficked fishers. Violence was quantitatively measured in eight papers with prevalence of: 11-26% in port convenience samples; 68-100% in post-trafficking service samples. Commercial fishers/seafarers whether trafficked or not worked extremely long hours; trafficked long-haul fishers had very limited access to care following injuries or illness. Lesser-known risks reported among fishers included penile oil injections and beriberi. We found just one work safety intervention study and inconclusive evidence for differences in the outcomes by nationality. Findings are limited by methodological weaknesses of primary studies.

**Conclusion:**

Results show an absence of high-quality epidemiological studies beyond sexual health. Formative and pilot intervention research on occupational, physical and mental health among GMS commercial fishers and seafarers is needed. Future studies should include questions about violence and exploitation. Ethical and reporting standards of grey literature should be improved.

**Trial Registration:**

Review registration number: PROSPERO 2014: CRD42014009656.

**Electronic supplementary material:**

The online version of this article (10.1186/s41256-018-0083-x) contains supplementary material, which is available to authorized users.

## Background

### Rationale

Commercial fishing at sea is among the world’s most dangerous occupations [1]. The Food and Agriculture Organization (FAO) estimates that Asians comprise 78% of 37.8 million working in capture fisheries globally [2], with China, the Philippines and Indonesia among the largest origin countries for an estimated 1.65 million seafarers worldwide [3]. Common occupational illnesses among seafarers include gastrointestinal, dental and dermatological conditions; and injuries to the extremities and back [4]. Accident risk is increased by inadequate use of protective gear, crew inexperience, and fatigue linked to insufficient manpower, long working hours and sleep deprivation, from noise and vibration of the boat, adverse weather conditions and night working or watch shifts [5]. However, most evidence on occupational health focuses on seafarers in or from developed regions in Europe, North America and Australasia. Fewer scientific studies have been conducted among Asian seafarers, whom may have different patterns of illness due to differences in work safety cultures, behaviours and diets [6].

Fishers and seafarers are vulnerable to exploitation and abuse, which is often exacerbated by their physical isolation in off-shore, mobile worksites. In recent years, the trafficking of fishers has emerged in the Greater Mekong Subregion (GMS) [7] as has the trafficking of seafarers in Central Asia [8]. GMS men and boys have been trafficked onto Thai fishing boats bound for Indonesian waters, from where large numbers of trafficked long-haul fishers have been repatriated reporting harsh working conditions, severe abuses and exploitation [7]. Because fishing transcends national borders, fishers face restrictions that require them to stay aboard vessels while in port, preventing escape and access to medical care [9].

Of existing systematic and non-systematic reviews on various health risks among commercial seafarers and fishers [5, 10–12]. none include studies on trafficked men. Furthermore, most reviews do not report the outcomes by nationality or migrant/citizen status, or by long or short-haul fishing, factors which may shape experiences of occupational hazards or health outcomes [13].

### Objectives

We sought to synthesize evidence on occupational health risks, physical, sexual and mental health problems and violence among fishers and seafarers from GMS countries (Cambodia, Laos, Myanmar, Thailand, Vietnam, China’s Yunnan province) working anywhere in the world, whom may be migrant/trafficked or working within their home country. While commercial fishing is the primary sector of interest in this review, seafaring is included because occupational health risks in seafaring can be considered similar to those in commercial fishing. Both sectors involve adverse working conditions in deep seas, with most work performed on open decks exposed to the elements, although fishing is more labour intensive, subject to less regulation than seafaring and has correspondingly higher mortality rates [11]. Long-haul fishing usually involves fishing trips lasting four weeks or more outside of territorial waters, whereas short-haul fishing refers to operations of four weeks or less within territorial waters [14].

This review scopes broad health outcomes, risks and associated factors, to provide an overview of existing health-related studies for this population. We reviewed all primary studies that measured the outcomes, risk behaviours and other factors affecting the outcomes. We compared findings by nationality and by long or short-haul fishing where data were available.

## Methods

### Search strategy

The review protocol is registered with the PROSPERO database of systematic reviews, registration number CRD42014009656. This review followed PRISMA guidelines (Additional file 1) [15]. A multi-stage search strategy was employed, comprising an electronic search of five databases (Embase, MEDLINE, Global Health, PsychoINFO and Academic Search Complete), using keywords and MESH/exploded terms (Additional file 2) for studies published between 1 January 1980 and 25 May 2016. We hand searched the International Maritime Health journal, and purposively searched the grey literature (e.g. reports from United Nations agencies) based on authors’ knowledge of previous studies on GMS migrant and trafficked fishers and seafarers.

### Selection criteria

Studies were eligible for inclusion if they: 1) included males or females from the GMS region working as commercial marine fishers/seafarers anywhere in the world; 2) measured the prevalence of any reported measure of occupational or physical health (e.g. risks/hazards, accidents/injuries/mortality, safety attitudes), sexual health (e.g. risk factors, diseases), mental health (e.g. disorders, suicide attempts), violence or treatment seeking behaviour for any health problem at either the workplace or individual level (method of assessing the outcomes is listed in Additional file 3); 3) presented results from peer or non-peer reviewed research based on either cross sectional surveys, cohort studies, experimental studies, qualitative studies or case studies (featuring interviews or focus groups). Grey literature, including technical reports and doctoral theses, were eligible. There were no language restrictions, or restrictions on methods used to measure the outcomes. When multiple eligible papers from the same study were identified, only the most definitive results were included for each relevant outcome. Each paper’s most definitive findings are reported separately in Tables S3-S6 in Additional file 4, but referenced in terms of the overall study in the discussion. The inclusion criteria initially included studies with fishers and seafarers of all Asia-Pacific nationalities and/or those fishing/seafaring anywhere in Asia-Pacific. Inclusion criteria were narrowed at the full-text screening stage to those above to yield a smaller number of regionally focused studies which would be more useful to service providers working with this population.

Studies were excluded if they: 1) did not include commercial marine GMS fishers/seafarers (e.g. studies with inland fishers, divers, traditional non-commercial fishers, leisure fishers were excluded); 2) did not present disaggregated data for the outcome measures where the eligible study population were included as a sub-group/data were unobtainable; 3) had study samples of fewer than five participants. Systematic and other reviews were not eligible for inclusion, although they were identified during title and abstract screening and used for the purposes of forwards/backwards citation tracking.

### Data extraction

Two reviewers (NP and LHN) screened downloaded titles and abstracts for potential inclusion; the same reviewers then assessed the full-text of potentially eligible papers against the inclusion criteria. If studies collected data on the study population as part of a larger sample, authors were contacted for relevant disaggregated data. An online data extraction form was developed and piloted by NP. Data from all included papers were extracted by NP; LHN independently extracted data from a random sample of 33% of included studies as a check; disagreements were resolved by discussion. We contacted nine authors for further information, five responded and provided disaggregated data by nationality as requested (*n*=2/5 of these studies were later excluded, one because it had a sample size of fewer than five participants and the second for not including the study population).

Data were extracted on study design, sample characteristics, the outcomes and definition and method of assessing the outcomes. Sample characteristics included whether participants were trafficked or forced labourers, as defined by participants, providers or researchers: no restrictions were placed on the method by which trafficking/forced labour status was assessed.

### Data analysis

We reported mainly prevalence, odds ratios and risk ratios for quantitative studies, focusing on adjusted analyses where available. Where possible, outcome measures were extracted separately by nationality and/or by long or short-haul fishing. Pooled estimates were not calculated for the outcomes due to heterogeneity in study sample selection, definitions, methods of assessing the outcomes and predominance of non-representative/convenience samples. Instead, we focused on describing the study results, limitations and implications. We did not use qualitative synthesis methods for qualitative studies. Instead, for qualitative studies on trafficked fishers of non-GMS nationalities, results were summarized for the whole sample with nationality/long or short-haul distinctions made clear where available in that study. Similarly, all results relevant to the outcomes of interest for this review were summarized for qualitative studies that included key informants.

### Quality appraisal

Methodological quality of studies was appraised independently by two reviewers; NP appraised all studies, while LHN appraised a randomly selected 33% of studies (*n*=11, the same studies for which data were extracted by LHN). We used the National Heart Lung and Brain Institute (NHLBI) quality assessment tool for quantitative studies [16] and the Critical Appraisal Skills Programme (CASP) for qualitative and mixed methods studies [17] (listed in Additional file 5). The NHLBI tool included 14 questions about study quality and the CASP tool included ten items. Reviewers were then asked to rate the study as Good, Fair or Poor based on their answers and to report the study’s limitations, as per guidance accompanying the NHLBI quality assessment tool. Reviewers were asked to make an overall qualitative judgement for these quality ratings, i.e. the ratings are not based on summary scores. We took this approach with the CASP checklist as well. Questions in both tools focus on sampling methods, sample characteristics, the participation rate and analysis method. Quality ratings (see Tables 1 and 2) and limitations were not used to exclude studies, but are referred to in the discussion. With predominantly cross-sectional studies included and considering the limitations of this study design, we use the term “quality” rather than risk of bias to indicate that studies/papers were assessed based on the best methodology the authors could offer for a cross-sectional study, rather than theoretical grounds for risk of bias [15]. We did not assign overall quality ratings for two grey literature reports where conducting a research study was not the explicit aim (i.e. investigative reports), but for which qualitative information was useful when discussing the remaining studies.

**Table 1:** Peer-reviewed papers on health from database searches (n=20)

Author (year)	Study design (year of data collection)	Sampling method	Sample description	Outcomes of interest	Country	Study quality
Entz et al. (2000)^a^ [18]	Cross-sectional survey (1998)	Convenience sampling at fishing ports	*N*=818 Fishermen (582 Thai, 137 Burmese, 99 Cambodian)	HIV/AIDS, Condom use, Alcohol/drug use	Thailand	Good
Entz et al. (2001)^a^ [19]	Cross-sectional survey (1998)	Convenience sampling at fishing ports	*N*=818 Fishermen (582 Thai, 137 Burmese, 99 Cambodian)	Sexual health, Treatment seeking behaviour	Thailand	Good
Nguyen et al. (2011)[27]	Cross-sectional survey (2007)	Purposive sampling via marine companies	*N*=94 Vietnamese seafarers	HIV/AIDS, Hepatitis B	Vietnam	Poor
Ford and Chamrathrithirong (2007)^b^ [20]	Cross-sectional baseline survey (2004)	Stratified, snowball sampling by occupational/ geographic groups	*N*=1603 Fishermen (1263 Burmese, 333 Cambodian)	Condom use	Thailand	Good
Ford and Chamrathrithirong (2008)^b^ [21]	Mixed methods study, cross-sectional baseline survey (2004) (study [20]), Qualitative interviews, focus groups (2007)	Stratified snowball sampling by occupational/geographic groups (quantitative), Purposive sampling (qualitative)	*N*=1603 Fishermen (1263 Burmese, 333 Cambodian), *N*=29 key informants, *N*=4 focus groups (5-7 Fishermen each)	Condom use, HIV/AIDS knowledge	Thailand	Good
Musumari and Chamchan (2016)* [22]	Cross-sectional baseline (2010), endline survey (2014)*	Stratified, snowball sampling by occupational/ geographic groups	Baseline: *N*=578 Fishermen (148 Myanmar, 430 Cambodian). Endline: *N*=510 Fishermen (125 Myanmar, 385 Cambodian)	Condom use, HIV/AIDS knowledge	Thailand	Good
MOPH (2011) [26]	Cross-sectional baseline survey (2003-5) for randomized trial	Consecutive sampling in *n*=47 health service provider screening sites	*N*=194 Thai fishermen (*N*=192 screened/tested for HIV)	HIV/AIDS	Thailand	Good
Sopheab et al. (2006) [24]	Cross-sectional household and individual survey (2002)	Stratified random cluster sampling	*N*=262 Cambodian fishermen	Condom use, Healthcare seeking behaviour	Cambodia	Fair
Ohnmar et al. (2009) [23]	Cross-sectional household survey (1999)	Random sampling	*N*=639 Burmese fishermen	Sexual health – penile practices, Condom use	Thailand	Good
Samnang et al. (2004) [25]	Cross-sectional survey (2000)	Convenience sampling	*N*=262 Cambodian fishermen	HIV/AIDS/Sexual health, Condom use, Alcohol use	Cambodia	Good
UNAIDs (1998) [28]	Cross-sectional survey, Qualitative in-depth interviews (year unclear)	Convenience sampling (seafarers/ fishermen), Purposive sampling (key informants)	*N*=110 Vietnamese seafarers/fishermen, *N*=173 Key informants	HIV/AIDS knowledge, Drug use, Treatment seeking behaviour	Vietnam	Poor
Levin et al. (2010)^c^^ [42]	Cross-sectional survey (2005)	Convenience sampling at fishing port	*N*=78 Fishermen (82% Vietnamese)	Occupational health – hours, work safety attitudes	USA	Fair
Carruth et al. (2010)^c^^ [43]	Focus groups, sampled from study [42] participants (year unclear)	Purposive sampling	*N*=3 Focus groups - 15 participants (9 Male, 6 Female, Vietnamese fishers/ key informants)	Occupational health - work safety attitudes	USA	Good
Levin et al. (2016)^d^^ [29]	Cross-sectional baseline (2008) endline (2012) surveys in prospective quasi-experimental community trial	Consecutive, convenience sampling (baseline), convenience sampling (endline) (3 sites/interventions)	Baseline: *N*=227 Fishers (97% Vietnamese, 86% Male). Endline: *N*=206 Fishermen (99.0% Vietnamese, 89% Male)	Occupational health – work safety attitudes, hypertension	USA	Fair
Levin et al. (2016)^d^^ [59]	Cross sectional survey (2008)	Consecutive, convenience sampling	*N*=227 Fishers (96.9% Vietnamese, 86% Male)	Occupational health - hearing loss	USA	Fair
Hansen et al. (2008) [44]	Secondary analysis of accident reporting data from 4 sources (2003)	NA, Administrative records	*N*=3253 Southeast Asian seafarers (668 Thai, 59 Vietnamese)	Occupational health - accidents	Denmark	Fair
Pe et al. (2005)^e^^ [40]	Cross-sectional household survey (2003)	Unclear (suggests every household sampled – could be census)	*N*=46 Sea snake bite victims (98% Fishermen)	Occupational health – sea snake bite, Treatment seeking behaviour, clinical symptoms	Myanmar	Fair/Poor
Pe et al. (2006)^e^^ [41]	Cross sectional household surveys (2003-4) (includes study [40])	Unclear (suggests every household sampled – could be census)	*N*=187 Sea snake bite victims (85% Fishermen)	Occupational health – sea snake bite, Treatment seeking behaviour, clinical symptoms	Myanmar	Fair/Poor
Doung-ngern et al. (2007) [35]	Cross-sectional survey, examination of medical records (2005)	Case series	*N*=28 Fishermen (4 Thai, 24 Burmese)	Occupational health – beriberi, clinical symptoms	Thailand	Fair
Kiss et al. (2015)^^ [31]	Cross-sectional survey (2011-13)	Prospective consecutive sampling in post-trafficking services	*N*=275 Fishermen (Trafficked, 217 Cambodian – 196 Long-haul, 55 Burmese/Short-haul, 2 Thai, 1 don’t know)**	Occupational health– hazards, injuries, Violence, Mental health Treatment seeking behaviour	Thailand, Cambodia	Good

^a^ same study

^b^ same study

^c^ same study. Percentage Vietnamese is assumed from percentage whose primary language is Vietnamese

^d^ same study. Percentage Vietnamese is assumed from percentage whose primary language is Vietnamese

^e^ same study

^Sample is not wholly comprised of GMS fishermen/seafarers, but includes high proportion of them in the sample

*disaggregated data for fishermen from baseline and end line surveys provided by Kathleen Ford (studies 113, 209)

^^Pocock and Zimmerman were co-authors in this study

**sole study among peer-reviewed health papers on trafficked fishers

**Table 2:** Papers from grey/non-health literature from purposive search (*n*=13)

Author (year)	Study design (year of data collection)	Sampling method	Sample	Outcomes of interest	Host country	Study quality
Robertson/IOM (2011) [36]	Qualitative in-depth interviews, focus groups (2008-9)	Purposive (via assistance organizations) and snowball sampling	*N*=8 Fishermen (Trafficked), *N*=9 Focus groups (6-15 Burmese/Cambodian fishermen each), *N*=63+ Key informants	Violence, Adverse conditions	Thailand	Fair
Brennan/Solidarity Centre (2009) [30]	Qualitative in-depth interviews (some quantitative findings) (2008)	Purposive sampling via assistance organization	*N*=25 Fishermen (some Trafficked, 24 Burmese, 1 Cambodian, 19 Short-haul, 11 Long-haul boats)	Violence, Adverse conditions, Mental health (qualitative description)	Thailand	Fair
UNIAP (2009) [46]	Qualitative in-depth interviews and case analysis (2007-8)	Purposive sampling via assistance organizations	*N*=49 Cambodian fishermen (Trafficked)	Violence, Adverse conditions	Thailand, Malaysia	Fair
Pearson/ILO (2006) [45]	Cross-sectional survey, Qualitative in-depth interviews (2005)	Snowball sampling (fishermen), Simple random sampling (employers)	*N*=21 Burmese fishermen (some Forced labour), *N*=40 Thai employers	Violence, Adverse conditions, Treatment seeking behaviour	Thailand	Fair
Fujita (2010) [56]	Cross-sectional household survey (2009)	Purposive sampling via assistance organization	*N*=19 Burmese fishermen households	Adverse conditions	Thailand	Poor/Fair
ILO/ARCM (2013) [14]	Cross-sectional survey (2012)	Stratified, multi-stage convenience sampling	*N*=596 Fishermen (some Forced Labour, 306 Burmese, 241 Cambodian, 49 Thai, 185 Short-haul, 106 Long-haul)	Occupational health – injuries, Violence, Adverse conditions	Thailand	Good
Baker/UNACT (2015) [39]	Cross-sectional surveys (2009, 2012, 2013)	Systematic random sampling	*N*=125 Cambodian fishermen (Deportees, some Trafficked)	Occupational health – work safety, Violence, Adverse conditions	Thailand, Cambodia	Good
Verite (2015) [34]	Qualitative in-depth interviews (year unclear)	Unclear/Not specified (could be purposive)	Unspecified no. Cambodian/Burmese fishermen (some Forced Labour), Key informants	Occupational health – hazards, Adverse conditions	Thailand	N/A
Yea (2014) [32]	Qualitative in-depth interviews, case file review (2010-14)	Unclear (could be purposive, based on 2012 study)	*N*=24 Fishermen (Trafficked, 3 Cambodian, 21 Filipino, 1 Indonesian) *N*=63 case files of Filipino fishermen	Occupational health – hazards, injuries, Adverse conditions Violence	Taiwan, Singapore	Fair
Day/HAGAR (2015) [37]	Qualitative in-depth interviews, focus groups (2014-15)	Purposive sampling via assistance organizations	*N*=8 Cambodian fishermen (Trafficked), *N*=1 Focus group (10 Cambodian fishermen), *N*=33 Key informants	Occupational health – injuries, Adverse conditions, Violence, Mental health (qualitative description)	Thailand, Indonesia, Malaysia	Fair/Good
EJF (2013) [52]	Qualitative in-depth interviews (2013)	Purposive sampling via assistance organization	*N*=6 Burmese fishermen (Trafficked)	Adverse conditions, Violence	Thailand	N/A
Stringer et al. (2016)^a^ [38]	Qualitative in-depth interviews (2011-14)	Purposive (via assistance organization) and snowball sampling	*N*=292 Fishermen (Forced Labour, from Indonesia, Myanmar, China, Philippines), Unspecified no. of Key informants	Occupational health – injuries, Adverse conditions, Violence	New Zealand	Fair/Good
Surtees (2014) [33]	Qualitative in-depth interviews, case file review (2013-14)	Purposive (via assistance organizations) and snowball sampling	*N*=11 Cambodian fishermen (Trafficked) *N*=20 case files of Cambodian fishermen, *N*=42 Key informants	Occupational health – hazards, injuries, Violence, Adverse conditions, Mental health (qualitative description)	South Africa	Fair/Good

^a^peer-reviewed non-health paper

## Results

**Fig. 1:**
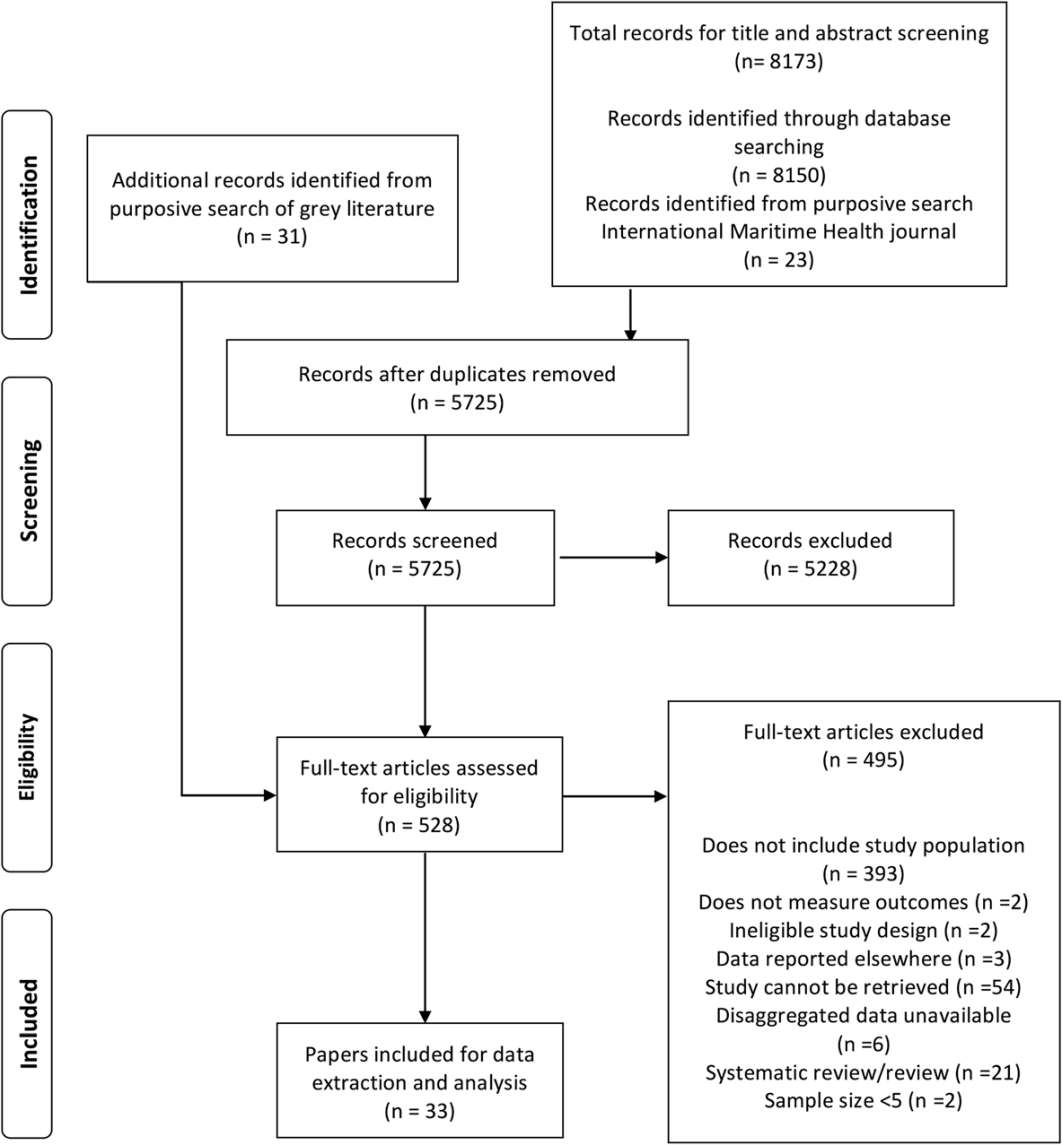
Flowchart of primary study selection

The study selection process is presented in Fig. 1. Including grey literature, our searches returned 5725 unique records, of which 5228 were excluded following title and abstract screening. Full-text copies of the remaining 528 papers that met or potentially met the inclusion criteria were retrieved. After full-text screening, 33 papers were retained for inclusion in the review. Of 33 papers, 20 were identified from database searches (Table 1) and 13 papers were identified from the purposive search of grey literature (Table 2). All of the included papers were published in English.

### Characteristics of included papers

Tables 1 and 2 summarize key features of the 33 included papers. Papers that report on the same studies are grouped together. The 33 papers reported on 27 studies, which were based in Thailand (*n*=14), Vietnam, Myanmar and Cambodia (*n*=2 respectively), the USA, Denmark and South Africa (*n*=1 respectively); four studies were conducted in multiple regions. Papers from peer reviewed literature (Table 1, *n*=20) examined health among citizen or migrant fishers/seafarers; 11 papers focused on sexual health, nine on occupational and physical health; one paper included mental health (Tables S3-S5 in Additional file 4). Thirteen grey literature papers focused on adverse conditions and exploitation in smaller samples (Table 2), twelve of which included trafficked or forced fishers/seafarers; eight papers (seven grey literature, one peer-reviewed) quantitatively measured violence among trafficked fishers/seafarers.

### HIV/AIDS/sexual health

Eight of 11 papers on HIV/AIDS/sexual health were of good quality and focused on risk factors for HIV (Additional file 4: Table S3). Six papers discussed migrant fishers’ risk behaviours in Thailand [18–23], while five papers discussed domestic fishers in Cambodia [24, 25], Thailand [26] and Vietnam [27, 28]. Among men who visited sex workers, condom use ranged from 75.6% [18] to 57% [24] although different timescales and populations hinder comparability. More frequent condom use with sex workers than with regular partners was linked to trust and 100% condom use policies in brothels among migrant fishers in Thailand [21]. Similarly, condom use with brothel-based sex workers (91.5%) was higher than with non-brothel based sex workers (70.0%) among fishers in Cambodia [25]. Ohnmar et al. found that younger age, low education, Mon ethnicity and being in Thailand for longer was associated with penile oil injections among migrant Burmese fishers [23]. Two studies reported that some fishers were injecting drug users which may be linked to HIV [18, 28]. Being a migrant fisher/seafarer was associated with increased odds of ever being tested for HIV in one study [22], which Ford and Chamratrithirong suggested was linked to knowing someone who’d died of AIDS [21]. One study reported greater descriptive/unadjusted improvements in AIDS knowledge and condom use among migrant Cambodian fishers than for Burmese fishers in Thailand following community based awareness raising interventions [22]. Entz et al. found higher proportions of Thai fishers ever had an STD compared to migrant fishers, likely due to inaccurate self-reports/reduced likelihood that migrant fishers could visit clinics for accurate diagnosis compared to Thais [19]. Migrant fishers also had higher odds of self-care for general health than Thai fishers [19].

### Occupational and physical health

Nine peer-reviewed papers described factors affecting occupational health, accidents/injuries, treatment seeking behaviour and physical health symptoms among fishers and seafarers; most papers (*n*=7/9) were fair or good quality (Table 1, Table S4 in Additional file 4). Grey literature which included trafficked fishers touched on similar themes; most was of fair quality (*n*=6/11) (Table 2, Table S6 in Additional file 4). Among Vietnamese fishers in the USA, 29.6% worked over 16 hours/day at baseline in an intervention study (Additional file 4: Table S4) [29]. In cross-sectional convenience/small samples of Burmese fishers in Thailand, long-haul fishers worked 18-24 hours/day, compared to 13-14 hours/day among short-haul fishers (some trafficked) [30] (Additional file 4: Table S6). In the International Labour Organization’s (ILO) large cross-sectional survey at Thai ports, 28.3% and 25.3% of long and short-haul fishers respectively worked 17-24 hours/day (some forced labour) [14]. In Kiss et al., 41.8% of trafficked fishers worked 20 or more hours/day every day [31]. Trafficked Cambodian fishers reported working hours of 18-22 hours/day in other studies (Additional file 4: Table S6) [32, 33].

Occupational hazards among trafficked fishers discussed in four papers included: long hours in sun/cold/wet without breaks (96.7%) [31], men being pulled overboard by heavy nets [34], falling overboard during storms and not being recovered [33], being forced to work in heavy storms and in cold storage and polar regions with no protective gear [32, 33]. Trafficked fishers commonly reported having no or bad safety/survival equipment [31], life jackets/buoys were unavailable or locked up (Additional file 4: Table S6) [32, 33]. Doung-ngern et al. described 53.6% (*n*=15/28) probable cases of beriberi (vitamin B1 deficiencies) on a ship comprised mainly of Burmese fishers [35]. Case fatality was high (13%, *n*=2/15). Among *n*=13 physically examined patients, 100% were hypertensive [35]. Studies with trafficked fishers noted inadequate drinking water and nutrition, due to the need to conserve supplies while on long-haul trips [33, 36]; rotten/expired food [32]; consumption of mainly raw food [37] and eating fish bait to survive [32, 38] Transshipment in long-haul fishing (the practice of refuelling boats/exchanging supplies at sea) saw trafficked fishers spend up to 3 years at sea without docking [33]. Men were tortured or sold to other boats for attempting escape [33, 37]. Conversely, another study of returning migrant Cambodian fishers from Thailand noted that most (72.8%) had free movement [39]. Pe et al. found high case fatality (11.2%) among sea snake bite victims in Myanmar [40, 41]. Bites were mainly incurred during fishing tasks and most victims self-treated or used traditional healers (Additional file 4: Table S4) [40, 41].

Three papers described attitudes towards work safety among older Vietnamese immigrant shrimp fishers in the USA (Additional file 4: Table S4) [29, 42, 43]. In a quasi-experimental community trial of work safety interventions, Levin et al. reported statistically and practically significant increases in safety attitudes for fatigue and noise interventions, but not winch safety [29]. Among returning migrant Cambodian fishers from Thailand, 63.2% reported safe working conditions (some trafficked) [39]. In the ILO survey at Thai ports, 91.9% reported being aware of safety risks in fishing; 20.6% were ever injured (some forced labour) (Additional file 4: Table S6) [14].

Hansen et al. found lower adjusted accident reporting rates among Southeast Asian seafarers (Incidence Rate Ratio (IRR) 0.29, Confidence Interval (CI):0.22-0.38) compared to Eastern (IRR 0.65, CI:0.50-0.85) and Western seafarers (reference) (Additional file 4: Table S4) [44]. Among fishers using post-trafficking services, half (49.8%) of Cambodians (mostly long-haul) experienced serious injuries compared to a third (36.4%) of Myanmar (mostly short-haul) fishers [31]; similarly, higher injuries prevalence was observed among long-haul (26.4%) compared to short-haul (19.4%) fishers in a convenience sample at Thai ports (Additional file 4: Table S6) [14]. Other studies with trafficked fishers discussed common injuries, including wounds from fishing hooks to the face, arms and neck lodged in the skin [32, 37], injuries from unguarded machinery [33] and lost limbs [31, 32]. Seriously sick/injured trafficked fishers were forced to wait until docking for medical care, some died waiting [32]; 36% of long-haul fishers in another study witnessed co-workers become sick and die at sea [30]. Another study of trafficked fishers found that injured men were forced to remain below deck when in port, and were denied medical assistance (Additional file 4: Table S6) [38].

Care among trafficked fishers was negligible; one study found that 96.9% of Myanmar (short-haul) fishers received no care, while 43.2% of Cambodian (long-haul) fishers reported receiving care from traffickers/employers [31]. Yea 2014 reported trafficked fishers’ wounds being stitched with no antiseptics or pain relief [32]. Several studies reported that inadequate/basic/expired medicines were given to trafficked fishers [30, 32, 36, 37]. Language barriers meant trafficked fishers couldn’t discuss health problems or medicines with superiors [33]. Among a small sample of Burmese fishers, half turned to each other for help when sick and a third turned to relatives (some forced labour) (Additional file 4: Table S6) [45]. Among trafficked fishers, Cambodians (long-haul) had worse physical health than Myanmar (short-haul) fishers, e.g. 30.9% and 10.9% respectively reported poor self-assessed health. Other health problems included chronic headaches, incorrectly or unhealed broken bones [33, 37], skin infections and persistent coughing [31, 33] (Additional file 4: Table S6).

### Mental health

One good quality study of trafficked fishers using post-trafficking services was included in the review. Kiss et al. described high prevalence of mental health disorders, particularly for Cambodian compared to Myanmar fishers (e.g. 63.0% and 21.8% were symptomatic of depression respectively) (Additional file 4: Table S5) [31]. Higher proportions of Cambodians experienced suicidal thoughts and attempted suicide compared to Myanmar fishers [31]. Despite high symptom prevalence, just 15.3% were concerned for their mental health [31]. Three grey literature papers whose samples included trafficked fishers discussed mental health (Additional file 4: Table S6) [30, 33, 37]. Long-haul (mainly Burmese) fishers who were regularly beaten considered suicide in one study [30]. Poor mental health from violence experienced or witnessed, with isolation at sea for extensive periods/delays in returning home compounded mental health problems among Cambodian fishers [33]. Anger, anxiety, stress, memory loss, aggression and substance misuse were observed among returned Cambodian fishers [33, 37].

Kiss et al. found that 33.6% of trafficked fishers were concerned about guilt or shame [31]; grey literature noted guilt/shame feelings linked to being a “failed migrant” without earnings, being deceived and/or being unable to protect oneself and pity from the community [37], although this may translate to feeling loved for some men [33]. Men feeling guilt/shame may avoid returning home and transfer to another boat hoping to earn money [33].

### Violence

Eight studies quantitatively measured violence but used different interview questions, hindering comparability but offering an overall indication of violence experienced. Two studies of solely trafficked Cambodian fishers found high prevalence of physical violence (93.5% and 100%) [33, 46]; with one study anecdotally suggesting that Cambodians were beaten more than other nationalities [33]. Conversely, being severely beaten was higher among Myanmar fishers than other nationalities in a convenience sample at ports (16.3% relative to 2.5% among Cambodians) [14], while another study reported higher prevalence of severe violence among Myanmar trafficked fishers (67.3%) relative to 50.2% among trafficked Cambodians [31]. Compared to trafficked fishers using post-trafficking services, three studies found lower prevalence of violence, e.g. 14% among Burmese fishers sampled at a port [45], to 50% among Burmese fishers being assisted by NGOs [30]; 26.3% of Cambodian fishers sampled at the Thai-Cambodia border said violence was a problem [39]. Among trafficked fishers, Stringer et al. reported that sexual abuse (groping, indecent exposure, rape) by superiors was common [38], compared to a low 1.8% prevalence of sexual violence in Kiss et al., which may be linked to reporting bias and/or only asking about forced sex (not other kinds of sexual abuse) [31]. Fishers in two studies reported that they were beaten for not working hard enough or when they were caught resting [33], made mistakes, or were tired [38]. Conversely, one investigative report using convenience sampling found that violence was uncommon and occurred mainly when men were drunk, overworked or when they lost their temper and fought at sea [34].

## Discussion

### Key findings

This review uncovered myriad health risks that GMS commercial fishers/seafarers face. Migrant fishers were likelier to self-treat compared to Thai fishers due to cultural, legal and linguistic barriers in accessing care [19]. The Thai government has since implemented a migrant health insurance program which aims to mitigate some of these barriers [47]. Penile oil injections, a lesser reported health risk among migrant fishers, leads to painful complications and highlights the need for targeted behavioural interventions. Among peer-reviewed studies, studies on HIV/AIDS/sexual health tended to be of higher quality compared to studies reporting other health risks, reflecting the dominance of policy concerns around HIV transmission among fishers to the general population.

Occupational risks were diverse. Commercial fishers worked long hours, with extreme hours observed among trafficked fishers, who have fewer/no breaks or respite between trips compared to non-trafficked men. Similarly, despite facing the same occupational hazards, trafficked fishers had limited to no access to protective gear. Lesser-known occupational risks were documented, including sea snake bites and vitamin B1 deficiencies leading to beriberi, as reported among trafficked fishers in Thailand recently [48]. High case fatality can be avoided if fishing companies ensure varied diets via frequent shipments of meat/vegetables, provision of unpolished rice and supplements [49]. Levin et al. highlighted the importance of culturally and literacy appropriate work safety interventions [29]. A captain’s leadership was important to influence deckhands during safety training, which should be: delivered in native languages at appropriate literacy levels; be hands on; conducted during off-season periods; end in completion certificates and be culturally appropriate e.g. bright T-shirts with safety messages [29, 43]. We found a paucity of safety intervention studies with GMS fishers/seafarers. Low accident reporting rates among Southeast Asian seafarers are suggestive of under-reporting, where crew may experience negative consequences (e.g. being medically signed off from duty, causing financial losses). We may need interventions to encourage accident reporting among Southeast Asian seafarers. In samples which included trafficked men, long-haul fishers reported injuries more frequently than short-haul fishers; transshipment led to treatment delays which put men at risk [32]. Preventive OSH interventions and adequate PPE are particularly important for long-haul fishers. Notably, no studies examined chronic disease prevalence or risk factors, except two studies which reported on hypertension [29, 35]. Physical health symptoms in two other studies with trafficked fishers indicated undiagnosed tuberculosis [31, 33]. A permanent ban on transshipment would alleviate the risk of being trafficked for extremely long periods and ensure that men who need care have a better chance of receiving it [48].

Economics and the regulatory environment affect occupational hazards experienced [50]. Increases in accident rates in the US Gulf of Maine indicated that some boats were taking longer trips with fewer crew, or taking more risks in tough financial times [51]. Similarly, in Thailand, economic pressures on operators and reluctance to modernise has led to a reliance on trafficked labour [52]. Future studies in the GMS could consider how the economic and regulatory environment affects hazards experienced.

We found just one peer-reviewed study on mental health among trafficked fishers [31], with none among GMS fishers who may not be trafficked. Grey literature studies with trafficked Cambodian fishers noted limited mental health services upon return [33]. A non-systematic review of seafarers mental health in worldwide fleets estimated that suicides comprised 13% of deaths due to illness [10], hinting at a mental health burden which is exacerbated by stressors including family separation, loneliness, fatigue, communication difficulties between multinational crews, limited recreation and sleep deprivation [53]. Among trafficked fishers, there is an urgent need to identify culturally appropriate interventions that can be implemented in low-resource environments with non-specialists. Pre-departure mental health screening could be conducted among seafarers and long-haul fishers for evidence generation and for practical reasons; pre-departure psychiatric assessment among Filipino seafarers may be related to low disembarkation rates for psychiatric problems compared to fishers of other nationalities [54]. Among seafarers and fishers generally, reduced hours, duty tours and increased shore leave could positively affect mental health.

Although definitional variations complicate interpretation, violence was only reported in studies with trafficked fishers. Elsewhere, violence accounted for 5% of injuries among seafarers from Denmark, Spain, Croatia, Finland and the Philippines [55]. Violence and exploitation questions should be included in studies with fishers/seafarers to enhance our understanding of their prevalence and associated risk factors.

We found inconclusive evidence for differences in the outcomes by nationality as just two studies reported adjusted analyses [18, 19, 44]. Descriptive analyses indicated that Cambodian trafficked fishers had more injuries and worse physical and mental health than Myanmar trafficked fishers [31], whom appeared to experience violence more frequently than Cambodians [14, 31]. Future studies should explore associations between nationality and health outcomes.

Many studies, particularly grey studies with trafficked fishers, did not report whether ethical approval had been obtained from Institutional Review Boards (IRB). Quality of grey literature was a concern; reporting of analysis methods was infrequent and presentation of results was suboptimal (e.g. raw survey data). Several studies were unclear about whether they used quantitative or qualitative designs [30, 46, 56]. There is a need for better reporting standards in NGO and grey literature, akin to Strengthening the Reporting of Observational Studies in Epidemiology (STROBE) criteria for observational studies [57]; following these standards would enhance readability. Many grey studies are commissioned by international agencies, whom should ensure external IRB approval and improved reporting.

### Future research priorities

This review highlighted limited evidence on GMS fishers/seafarers which is needed to inform appropriate interventions; while a growing literature examines Chinese and Filipino seafarers’ health, more epidemiological studies should be conducted with other Asian samples, alongside building data collection and management capacity in recent origin countries [58]. Potential data collection points include: pre-departure medical examinations (chronic conditions); radio medical records at sea (accidents, illnesses); port-based clinics/hospitals or repatriation services (acute injuries/illness); shore-based sources (death certificates, hospital records, census returns where occupation is declared) [58]. Researchers in maritime health should lead efforts to validate appropriate instruments to improve study comparability. Studies should include questions on nationality and whether fishers are long or short-haul.

### Limitations of the review

The search strategy omitted citation tracking and contacting experts due to limited resources. Studies from developing countries are less likely to be indexed in international databases which may bias our results. Methodological problems of primary studies limit conclusions that can be drawn. Most studies used convenience or purposive sampling in cross-sectional designs, many didn’t explain rationale for sample size and did not discuss sample representativeness. Heterogeneity of measurement of outcomes (e.g. violence) hindered comparability. Differing operational definitions of trafficking and forced labour hinders interpretation of findings. More extreme cases of violence and poor health were found among post-trafficking service users compared to convenience samples of fishers recruited at ports. Yet, many trafficked persons do not access services. Moreover, the proportion of trafficked fishers/seafarers that were included in studies conducted outside of post-trafficking services is unclear. An indication of the “true” situation of trafficked GMS fishers may therefore lie in-between figures reported for fishers using post-trafficking services and those sampled at ports, in this review. Grey literature findings were mainly qualitatively described; it was not clear whether validated questions were used to assess the outcomes reported in quantitative grey studies. Many peer-reviewed studies gave limited information about study instruments. Most research findings and all of those from grey literature were descriptive; information continues to be lacking on demographic and other factors associated with poor health outcomes that could inform interventions targeting fishers’ health.

### Conclusion

There is a significant evidence gap on occupational, physical and mental health problems among GMS fishers, despite high levels of need for such evidence. Formative research and pilot intervention studies on culturally appropriate interventions is needed, especially for work safety among long-haul fishers who face delays in reaching shore following accidents/injuries. We should not need an HIV epidemic or trade sanctions for forced labour to care about fishers’ health.

## Additional files

Additional file 1: PRISMA Checklist. (PDF 76 kb)

Additional file 2: EMBASE search terms. (PDF 24 kb)

Additional file 3: Method of assessing the outcomes. (PDF 98 kb)

Additional file 4: Data tables. (PDF 133 kb)

Additional file 5: Quality Appraisal Tools. National Heart Lung & Brain Institute (NHLBI) Quality Assessment Tool for Observational Cohort and Cross-­-Sectional Studies. (PDF 65 kb)

## Abbreviations

CASP: Critical Appraisal Skills Programme
FAO: Food and Agriculture Organization
GMS: Greater Mekong Subregion
ILO: International Labour Organization
NHLBI: National Heart Lung and Brain Institute
OSH: Occupational Safety and Health
STROBE: Strengthening the Reporting of Observational Studies in Epidemiology
