# Supplementary material for: Occupational, physical, sexual and mental health and violence among migrant and trafficked commercial fishers and seafarers from the Greater Mekong Subregion (GMS): systematic review
Source: Glob Health Res Policy. 2018 Oct 1;3:28. doi: 10.1186/s41256-018-0083-x (PMC6166293; doi:10.1186/s41256-018-0083-x)
Supplement: Supplementary file 2 — EMBASE search terms. (PDF 24 kb) [file 41256_2018_83_MOESM2_ESM.pdf]

EMBASE search terms

1. (international OR nationali* OR foreign OR immigra* OR migra* OR traffick* OR slavery OR slave* OR forced labo?r OR labo?r exploitation OR exploit* OR mobile OR commercial OR non-European*) adj2 (fishing OR fisher* OR fisherm#n)
2. (international OR nationali* OR foreign OR immigra* OR migra* OR traffick* OR slavery OR slave* OR forced labo?r OR labo?r exploitation OR exploit* OR mobile OR merchant OR commercial OR non-European*) adj2 (seafar* OR seam#n OR boatm#n OR sailor OR navy OR naval)
3. 1 OR 2
4. fishing OR fisher* OR fisherm#n OR seafarer* OR seam#n OR boatm#n
5. Asia* OR Asia-Pacific OR Asia Pacific OR (Asia and Pacific) OR Southeast Asia* OR (South East Asia) OR Australia* OR Bangladesh* OR Bengali OR Bhutan OR Brunei OR Brunei Darussalam OR Burma OR Burmese OR Myanmar* OR Cambodia* OR Khmer OR China OR Chinese OR Hong Kong OR India* OR Indonesia* OR Japan* OR Korea* OR Lao* OR Lao* Peoples Democratic Republic OR Lao* PDR OR Malaysia* OR Malay OR Malacca OR Mongolia* OR Nepal* OR New Zealand OR Papua New Guinea* OR Philippines OR Filipin* OR Singapore* OR Sri Lanka* OR Thailand OR Thai OR Timor Leste OR Timor-Leste OR Vietnam* OR Viet Nam OR Taiwan* OR Pakistan* OR Cook islands OR Micronesia* OR Fiji* OR Kiribati* OR Marshall islands OR Nauru OR Niue OR Palau OR Samoa* OR Soloman islands OR Tonga* OR Tuvalu OR Vanuatu
6. 4 AND 5
7. 3 OR 6
8. exp health/ OR exp disease/
9. exp injury/ OR exp pain/
10. exp occupational disease/ OR exp occupational exposure/ OR exp occupational accident/ OR exp occupational hazard/ OR exp occupational health/ OR exp occupational safety/ OR exp occupational medicine/
11. exp fatality/ OR exp death/ OR exp homicide/
12. exp suicide/ OR exp automutilation/
13. exp sexual health/ OR exp sexually transmitted disease/ OR exp sexual behavio?r/
14. exp mental health/ OR exp mental disease/
15. exp anxiety/ OR exp depression/ OR exp hostility/ OR exp posttraumatic stress disorder/ OR exp psychosis/
16. exp fear/ OR exp guilt/ OR exp shame/
17. exp violence/ OR exp assault/ OR exp abuse/ OR exp rape/
18. (occupational OR workplace OR work OR maritime) adj1 (health OR risk OR safety OR medicine OR hazard OR disease OR exposure OR injur* OR accident*)
19. injur* OR accident* OR fatigue OR fatalit* OR wound* OR trauma*
20. Suicid* OR homicid* OR self-harm OR (self harm)
21. Health OR disease* OR illness* OR infect* OR syndrome* OR disabilit*
22. (sexual health) OR (sexually transmitted disease*) OR (sexually transmitted infection*) OR condom* OR contracept* OR (penile implant*) OR HIV OR AIDS
23. (Mental* OR psychosocial) adj2 (problem* OR disorder* OR ill* OR health OR stress* OR wellbeing OR well-being)
24. Anxiety OR depress* OR psychiatric OR bipolar OR psychos* OR schizophren* OR fear OR guilt OR hostil* OR shame
25. (post-traumatic stress) OR (posttraumatic stress) OR (post traumatic stress) adj1 disorder*
26. Violen* OR abus* OR assault* OR depriv* OR neglect OR rape
27. OR/8-26
28. 7 AND 27
29. molecul* OR membrane* OR cell* OR protein* OR pesticide* OR biomarker* OR biodivers* OR species OR multispecies
30. 28 not 29
