# Supplementary material for: Occupational, physical, sexual and mental health and violence among migrant and trafficked commercial fishers and seafarers from the Greater Mekong Subregion (GMS): systematic review
Source: Glob Health Res Policy. 2018 Oct 1;3:28. doi: 10.1186/s41256-018-0083-x (PMC6166293; doi:10.1186/s41256-018-0083-x)
Supplement: Supplementary file 3 — Method of assessing the outcomes. (PDF 98 kb) [file 41256_2018_83_MOESM3_ESM.pdf]

Supplementary file 3: Method of assessing the outcomes

Table 1b. Peer-reviewed papers on health from database search (n=20)

Author (year)	Outcomes of interest	Method of assessing outcomes
Entz et al. (2000)[a] [18]	HIV/AIDS,Condom use, Alcohol/drug use	HIV status assessed using oral fluid testing (immunochromatography assay kit/Western Blot). Standardized questions on sexual health/healthcare seeking behaviour
Entz et al. (2001)[a] [19]	Sexual health, Treatment seeking behaviour	HIV status assessed using oral fluid testing (immunochromatography assay kit/Western Blot). Standardized questions on sexual health/healthcare seeking behaviour
Nguyen et al. (2011) [27]	HIV/AIDS, Hepatitis B	HIV/Hepatitis B assessed using serological testing (immunochromatography assay kit/Western Blot)
Ford and Chamrathrithirong (2007)[b] [20]	Condom use	Standardized questions for condom use based on core indicators of UN General Assembly Special Session on HIV/AIDS and Global Fund to Fight AIDS, Tuberculosis and Malaria
Ford and Chamrathrithirong (2008)[b] [21]	Condom use, HIV/AIDS knowledge	Standardized questions for HIV/AIDS knowledge/condom use based on core indicators of UN General Assembly Special Session on HIV/AIDS and Global Fund to Fight AIDS, Tuberculosis and Malaria. Open-ended questions on ecological factors for HIV/AIDS risk behaviour for qualitative component.
Musumari and Chamchan (2016)[c] [22]	Condom use, HIV/AIDS knowledge	Standardized questions for HIV/AIDS knowledge/condom use based on core indicators of UN General Assembly Special Session on HIV/AIDS and Global Fund to Fight AIDS, Tuberculosis and Malaria
MOPH (2011) [26]	HIV/AIDS	HIV assessed using serological testing (EIA/Western Blot)
Sopheab et al. (2006) [24]	Condom use, Healthcare seeking behaviour	Standardized questions on condom use/healthcare seeking behaviour
Ohnmar et al. (2009) [23]	Sexual health – penile practices, Condom use	Standardized questions on condom use/sexual health/penile injections/implants
Samnang et al. (2004) [25]	HIV/AIDS/Sexual health, Condom use, Alcohol use	HIV assessed using serological testing (2x rapid immunosorbent assays). STI history as diagnosed by medical/health professional, condom/alcohol use by standardized questions
UNAIDs (1998) [28]	HIV/AIDS knowledge, Drug use, Treatment seeking behaviour	Non-standardized, non-validated questions for all outcomes
Levin et al. (2010)[d,e] [42]	Occupational health – hours, work safety attitudes	Standardized questions on work tasks, safety beliefs, self-reports of ability to use safety equipment/apply procedures aboard vessel, OSH training preferences
Carruth et al. (2010)[d,e] [43]	Occupational health - work safety attitudes	Open-ended questions on perceptions of risk, influences impacting safety on the vessels, training preferences

Levin et al. (2016)[d,e] [29]	Occupational health – work safety attitudes, hypertension	Standardized, validated questions based on Theory of Planned Behaviour framework, assessing behavioural, normative, control beliefs, intention for risk area on 6-point scale. Hypertension measured using single sitting automated blood pressure measurement
Levin et al. (2016)[d,e] [59]	Occupational health - hearing loss	Hearing loss assessed by audiometric tests
Hansen et al. (2008) [44]	Occupational health - accidents	Accidents as reported in: accident reporting forms/case files, insurance records, medical records
Pe et al. (2005)[e,f] [40]	Occupational health – sea snake bite, Treatment seeking behaviour, clinical symptoms	Structured questions on circumstances of sea snake bite, fatality, treatment-seeking behaviour, use of first-aid and prophylaxis
Pe et al. (2006)[e,f] [41]	Occupational health – sea snake bite, Treatment seeking behaviour, clinical symptoms	Structured questions on circumstances of sea snake bite, fatality, treatment-seeking behaviour, use of first-aid and prophylaxis
Doung-ngern et al. (2007) [35]	Occupational health – beriberi, clinical symptoms	Standardized questions on clinical symptoms, job responsibilities, daily activities on vessel, diet. Review of medical records, serological testing for thiamine deficiency (n=3 samples)
Kiss et al. (2015) [g] [31]	Occupational health– hazards, injuries, Violence, Mental health Treatment seeking behaviour	Violence: standardised, non-validated questions. Physical health: adapted version of the Miller Abuse Physical Symptoms and Injury Survey. Mental health: Hopkins Symptom Checklist 25 (depression, anxiety), Harvard Trauma Questionnaire (PTSD)

a. same study

b. same study

c. disaggregated data for fishermen from baseline and end line surveys provided by Kathleen Ford

d. same study. Percentage Vietnamese is assumed from percentage whose primary language is Vietnamese

e. sample is not wholly comprised of GMS fishermen/seafarers, but includes high proportion of them in the sample

f. same study

g. Pocock and Zimmerman were co-authors in this study

Table 2b. Papers from grey/non-health literature from purposive search (n=13)

Author (year)	Outcomes of interest	Definition of trafficking
Robertson/IOM (2011) [36]	Violence, Adverse conditions	Assistance provider identified/ Self-defined
Brennan/ Solidarity Centre (2009) [30]	Violence, Adverse conditions, Mental health (qualitative description)	Assistance provider identified
UNIAP (2009) [46]	Violence, Adverse conditions	Assistance provider identified
Pearson/ ILO (2006) [45]	Violence, Adverse conditions, Treatment seeking behaviour	Self-defined (Forced labour, ILO definition)
Fujita (2010) [56]	Adverse conditions	N/A
ILO/ARCM (2013) [14]	Occupational health – injuries, Violence, Adverse conditions	Self-defined (Forced labour, ILO definition)
Baker/ UNACT (2015) [39]	Occupational health – work safety, Violence, Adverse conditions	Self-defined (exploited at workplace and coerced or deceived in recruitment)
Verite (2015) [34]	Occupational health – hazards, Adverse conditions	Self-defined (Forced labour, ILO definition)
Yea (2014) [32]	Occupational health – hazards, injuries, Adverse conditions, Violence	Self-defined (exploited at workplace and coerced or deceived in recruitment)
Day/ HAGAR (2015) [37]	Occupational health – injuries, Adverse conditions, Violence, Mental health (qualitative description)	Assistance provider identified
EJF (2013) [52]	Adverse conditions, Violence	Assistance provider (governmental) identified
Stringer et al. (2016)[h] [38]	Occupational health – injuries, Adverse conditions, Violence	Assistance provider identified/ self-defined (ILO/European Commission definition)
Surtees (2014) [33]	Occupational health – hazards, injuries, Violence, Adverse conditions, Mental health (qualitative description)	Assistance provider identified/ self-defined (exploited at workplace and coerced or deceived in recruitment)

h. peer-reviewed non-health paper
