# Supplementary material for: Occupational, physical, sexual and mental health and violence among migrant and trafficked commercial fishers and seafarers from the Greater Mekong Subregion (GMS): systematic review
Source: Glob Health Res Policy. 2018 Oct 1;3:28. doi: 10.1186/s41256-018-0083-x (PMC6166293; doi:10.1186/s41256-018-0083-x)
Supplement: Supplementary file 4 — Data tables. (PDF 133 kb) [file 41256_2018_83_MOESM4_ESM.pdf]

**Supplementary File 4. Data tables**

## Table 3. HIV/AIDs and sexual health among commercial fishers and seafarers from GMS countries (n=11)

| Author (year) | Main findings |
|---|---|
| Entz (2000)[a] [18] | • Consistent condom use with sex workers/past year (75.6%); Injecting drug users (2.7%)<br>• Frequency of alcohol/drug use before/during sex: Never (40.0%), Sometimes (38.5%), Always (21.5%)<br>• Nationality association with HIV prevalence: Thai (14.6%) (reference), Burmese (16.1%) (UOR 1.12, CI:0.67-1.86), Cambodian (20.2%) (UOR 1.48, CI:0.86-2.55)<br>• Factors associated with HIV prevalence (multivariable): 25-32y/o, =>6 visits to sex workers, unmarried, tattooed |
| Entz (2001)[a] [19] | • History of STD (30.0%); Self-treatment of last STD (31.0%); Self-treatment of general health while ashore (32.0%)<br>• Ever had STD: Thai (35.5%) (reference), Burmese (11.1%) (AOR 0.54, CI:0.29-1.01), Cambodian (23.5%) (AOR 0.77, CI:0.45-1.32)<br>• Self-care of last STD: Thai (27.8%) (reference), Cambodian (30.4%) (AOR 0.84, CI:0.32-2.24), Burmese (66.7%) (AOR 4.07, CI:1.30-12.77)<br>• Self-care of general health while ashore: Thai (27.1%) (reference), Burmese (44.5%) (AOR 1.98, CI:1.33-2.95), Cambodian (46.5%) (AOR 2.21, CI:1.40-3.48) |
| Nguyen (2011) [27] | • Hepatitis B prevalence (54.3%) (HBsAg 9.6% + anti-HBs 44.7%); HIV prevalence (0%) |
| Ford (2007)[b] [20] | • No association with being a fisherman and using condoms with sex workers (AOR 0.88, CI:0.12-6.52) (other sectors is reference) |
| Ford (2008)[b] [21] | • AIDS knowledge score 0-15 (mean): 8.0. Fishermen had higher knowledge scores than male migrants in other sectors<br>• Condom use (mean): sex worker (3.6, range 1-4), regular partner (0.1, range 0-1), non-regular partner (2.0, range 1-4)<br>• Fishermen were more likely to visit sex workers, have non-regular partners, less likely to have regular partners Vs. other sectors. Condom use was similar<br>• Seafarers feel vulnerable to HIV, seen co-workers dying with no medical assistance on boats, aware that visiting sex workers makes them vulnerable to HIV<br>• Condom use usually 100% at brothels due to policy, less in other settings. With girlfriends/"love" relationships, condoms often not used<br>• Alcohol can give seafarers courage to visit sex workers but reduces likelihood of condom use<br>• Men aware of HIV testing sites, attend when feeling susceptible to HIV due to many sex partners. Some avoid going fearing positive result/time/money concerns<br>• Treatment by co-workers: HIV positive seafarers allowed to work while healthy/strong but looked down on by coworkers/community for having too many partners |
| Musumari (2016)[c] [22] | • AIDS knowledge score**, baseline (mean): Myanmar (13.87, CI:13.15-14.59), Cambodia (13.84, CI:12.96-14.72)<br>• AIDS knowledge score**, endline (mean): Myanmar (13.55, CI:13.11-13.98), Cambodia (16.26, CI:16.02-16.50)<br>• Condom use at last sex with regular partner, baseline: Myanmar (6.4%), Cambodia (2.6%), endline: Myanmar (4.8%), Cambodia (13.3%)<br>• Fishing sector significantly associated with ever testing for HIV (AOR, 2.51, CI:1.28–4.92) relative to other sectors |
| MOPH (2011) [26] | • HIV prevalence 5.24% (CI:3.62-6.85), AOR 4.82 (CI:2.48-9.39) (relative to other sectors, office/factory worker is reference) |
| Sopheab (2006) [24] | • Always condom use past 3 months sex workers (57%); Always condom use past 3 months girlfriend (17%); Condom use last sex with girlfriend (68%)<br>• Had STI treated at public or private clinic/hospital (75%) |
| Ohnmar (2009) [23] | • Penile oil injection prevalence (7.5%); Penile implant prevalence (12.4%); Condom use with CSWs: Penile oil injection/implant users (32.9%), Others (44.5%)<br>• Penile practices associated with sex with commercial sex workers (CSWs): No penile practice (32.2%) (reference), Penile oil injection (70.8%) (AOR 3.03, CI: 1.39-6.60), Penile implants (57.0%) (AOR 2.18, CI: 1.19-3.99)<br>• Factors associated with penile oil injections: younger age, low education, Mon ethnicity, +1year Thai residence. Penile implants associated +1year Thai residence |

| Author (year) | Main findings |
|---|---|
| Samnang (2004) [25] | • HIV prevalence (16.2%); Among 18-22y/o (12.8%), 23-29 y/o (20.3%), >29y/o (15.0%); STI diagnosed in past year (24.0%)<br>• Condom use: Brothel sex workers (91.5%), Non-brothel sex workers (70.0%)<br>• Condom use/sex workers HIV association: Always (47.8%) (reference), Frequently (24.3%) (AOR 1.04, CI: 0.5-2.3), Sometimes (23.5%) (AOR 0.8, CI: 0.3-1.8)<br>• Alcohol consumption past 12 months (79.4%); 3-4 days/week (10.0%), 1-2 days/week (11.5%), <1 day/week (25.5%), 3-4 times/month (10.5%) |
| UNAIDS (1998) [28] | • Seafarers/fishermen engage in commercial sex, have limited knowledge of HIV/AIDS. Comradeship on board is essential for survival and completing work tasks<br>• Some seafarers/fishermen are injecting drug users. Some share injecting equipment because of limited finances despite being aware of risk of HIV transmission. Peer pressure, depression, family problems are push factors for drug use<br>• Health workers, pharmacists not very knowledgeable about HIV/AIDS or transmission risks, don't actively promote condom use among seafarers. Administrators more knowledgeable. Seafarers/fishermen self-treat, seek advice from friends/purchase medicine at private pharmacies |

a. same study

b. same study

c. disaggregated data for fishermen from baseline and end line surveys provided by Kathleen Ford

**AIDS knowledge score range 0-20

### Table 4. Occupational and physical health among commercial fishers and seafarers from GMS countries (n=9)

| Author (year) | Main findings |
|---|---|
| Levin (2010)[d,e] [42] | • Fishing >12 hours/day (87.2%); Considers the job very safe, safe or neutral (70.5%); Receives safety training every year (59.0%) |
| Carruth (2010)[d,e] [43] | • Work requires physical and mental prowess and endurance, Fishermen usually spend 3 weeks at sea, night fishing<br>• Deaths from trauma, drowning, violence; Disability from physical injury. Alcohol consumption contributes to accidents, violence<br>• Experience/ability to anticipate safety risks e.g. mechanical failures are important. Captain's leadership skills are essential. Adverse events function of experience, training/knowledge, leadership, overconfidence, carelessness, rushing, poor judgement. Safety is tied to paying attention, awareness, discipline<br>• Influential captains earn deckhands' respect, which is necessary for captains to train them. Safety training should be in Vietnamese, based on deckhands' literacy level, convenient (off-season periods), hands on/practical (drills), end in completion certificates and target captains first |
| Levin (2016)[d,e] [29] | **Work > 16 hours/day**: Baseline (29.6%); Endline (40.0%); **Hypertension:** Baseline, >50% stage 1 or greater hypertension<br>**Winch safety intervention site+**<br>• Being careful prevents injury: pre (5.59) post (5.64); Injuries from becoming caught in machinery are possible to prevent: pre (5.45) post (5.43); I can prevent injuries by being aware: pre (5.60) post (5.70); I can prevent injuries by what I wear: pre (5.43) post (5.60)<br>**Hearing intervention site+**<br>• Wearing ear plugs while working on vessel will protect my hearing: pre (4.66) post (5.58)**; Hearing loss from noise exposure while working on vessel is often possible to prevent: pre (4.83) post (5.45)*; I can prevent hearing loss from noise exposure while working on vessel by wearing ear plugs: pre (4.75) post (5.79)**<br>**Fatigue intervention site+**<br>• Enough rest prevents injuries: pre (5.27) post (5.79)*; Injuries related to fatigue are possible to prevent: pre (5.15) post (5.88)**; I can prevent injuries while working aboard vessel by working fewer than 12 hours and getting enough sleep: pre (4.60) post (5.95)*** |
| Levin (2016)[d,e] [59] | • Hearing impaired prevalence* (59.4%). Associated with >15 years experience (AOR 2.04, CI:0.98-4.21), aged =>50 y/o (AOR 2.23, CI:1.07-4.63)<br>• Noise-Induced Hearing Loss prevalence* (53.8%). Associated with >15 years experience (AOR 2.23, CI:1.08-4.63), not aged =>50 y/o (AOR 1.37, CI:0.65-2.87) |
| Hansen (2008) [44] | • Accident prevalence: Thai (0.3%), Vietnamese (6.8%); Accident rate per 1000 years/sea: West Europeans (106), Southeast Asians (41), East Europeans (89)<br>• Crude incidence rate ratio: Western Europeans (1), Southeast Asians (0.38), Eastern Europeans (0.88)<br>• Adjusted incidence rate ratio: Western Europeans (1), Southeast Asians (IRR 0.29, CI:0.22-0.38), Eastern Europeans (IRR 0.65, CI:0.50-0.85) |
| Pe (2005)[e,f] [40] | • Cumulative incidence 1999-2002: 75.15/100,000; Case fatality 4.3%; 70% of victims bitten on hands, usually deep sea fishermen while drawing/unloading fishing nets and sorting fish; 30% bitten on legs, usually shallow water fishermen while setting up/drawing fishing nets in sea<br>• Healthcare seeking behaviour: local traditional healers (56.5%), hospitals (19.6%), clinics (17.4%), home treatment (6.5%)<br>• Clinical symptoms: drowsiness (87.0%), limb muscle ache (74.0%), limb muscle stiffness (80.0%), heavy upper eyelids (84.0%), dark urine (71.0%) |
| Pe (2006)[e,f] [41] | • Cumulative incidence varies from 75.15, 118.9, 318/100,000 over 1999-2003; Case fatality 11.2% across 4 sites<br>• 86.6% bitten during fishing activities; particularly drawing or using conical nets (34.7%), stake nets (19.3%), sorting fish (11.8%)<br>• Healthcare seeking behaviour: home treatment (44.3%), local traditional healers (37.9%), hospitals (9.0%), clinics (6.4%), no treatment (2.1%)<br>• Clinical symptoms: drowsiness (78.6%), muscle ache (71.6%), muscle stiffness (62.5%), heavy upper eyelids (56.6%), dark urine (31.5%) |
| Doung-ngern (2007) [35] | • Probable cases of beriberi: 53.6% (15 cases total, 14 cases Burmese, 1 Thai). Case fatality: 13% (2 deaths on board) |

| Author (year) | Main findings |
| --- | --- |
| | • Clinical symptoms: edema (60%), chest discomfort (54%) dyspnea (27%); Among N=13 physically examined patients (includes deceased), 100% hypertensive<br>• Diet: Fish and white rice only for 2 months prior to onset of symptoms; Total time at sea: 18 months (includes 5-month delay in docking) |
| Kiss (2015) [31] | **Occupational hazards**<br>• Worked =>20 hours/day: All (41.8%), Cambodian (48.8%), Myanmar (16.4%); Worked every day: 97.1%; No time off for sickness/holiday: 86.9%<br>• Long hours in sun/cold/wet without breaks: 96.7%; Small/unstable/badly maintained fishing vessel: 34.2%; Badly maintained or no fishing equipment: 28.0%<br>• No safety/bad/no survival equipment: All (61.8%), Cambodian (54.4%), Myanmar (92.7%); No personal protective equipment: 13.5%<br>**Injuries/healthcare**<br>• Injured at least once: All (46.6%), Cambodian (49.8%), Myanmar (36.4%); Injuries still cause pain/difficulty: All (51.6%), Cambodian (57.4%), Myanmar (20.0%)<br>• Deep/very long cut: All (79.3%), Cambodian (73.2%), Myanmar (40.0%); Serious head injury: All (20.5%), Cambodian (23.4%), Myanmar (5.0%); Back/neck injury: All (36.2%), Cambodian (43.0%), Myanmar (0.0%); Lost body part: All (4.7%), Cambodian (5.6%), Myanmar (0.0%)<br>• Care, Doctor/nurse: All (7.9%), Cambodian (8.9%), Myanmar (3.1%); Trafficker/employer: All (36.5%), Cambodian (43.2%), Myanmar (3.1%); Coworker: All (7.3%), Cambodian (8.9%), Myanmar (0.0%); Traditional healer: All (2.3%), Cambodian (8.9%), Myanmar (0.0%); No care: (52.3%), Cambodian (41.8%), Myanmar (96.9%)<br>**Physical health**<br>• Headaches: All (28.4%), Cambodian (32.7%), Myanmar (12.7%); Skin problems: All (18.6%), Cambodian (20.3%), Myanmar (12.7%); Weight loss: All (22.9%), Cambodian (27.2%), Myanmar (5.5%); Persistent cough: All (13.8%), Cambodian (15.7%), Myanmar (3.6%); =>3 areas/pain: All (29.1%), Cambodian (34.6%), Myanmar (9.1%); Poor self-assessed health: All (26.9%), Cambodian (30.9%), Myanmar (10.9%); Physical health concerns: All (33.6%), Cambodian (36.6%), Myanmar (23.7%)<br>**Violence**<br>• Physical violence: All (68.4%) Cambodian (65.4%), Myanmar (80.0%); Sexual violence: All (1.8%), Cambodian (1.8%), Myanmar (1.8%)<br>• Severe violence: All (53.8%), Cambodian (50.2%), Myanmar (67.3%); Less severe violence: All (17.5%), Cambodian (18.9%), Myanmar (12.7%)& |

d. same study. Percentage Vietnamese is assumed from percentage whose primary language is Vietnamese

e. sample is not wholly comprised of GMS fishermen/seafarers, but includes high proportion of them in the sample

f. same study

+Mean scores in brackets on Likert scale 1-6 from strongly disagree/unlikely (1) to strongly agree/likely (6) *p<0.05 **p<0.01 ***p<0.001

&Severe violence: being kicked, dragged or beaten up; tied or chained; choked or burned; released a dog to bite or scratch; being threatened with a weapon; cut with a knife, shot or forced to have sex. Less severe violence: being slapped, pushed, hit with fist

**Table 5. Mental health among commercial fishers and seafarers from GMS countries (n=1)**

| Author (year) | Main findings |
|---|---|
| Kiss (2015) [31] | • Depression: All (54.4%), Cambodian (63.0%), Myanmar (21.8%); PTSD: All (39.4%), Cambodian (46.8%), Myanmar (10.9%); Anxiety: All (44.9%), Cambodian (55.6%), Myanmar (5.5%)*; Suicidal thoughts: All (7.3%), Cambodian (8.8%), Myanmar (1.8%); Suicide attempts past month: All (4.4%), Cambodian (5.6%), Myanmar (0%)<br>• Concerned for Mental health: All (15.3%), Cambodian (17.6%) Myanmar (7.3%); Guilt or shame: All (33.6%), Cambodian (34.3%), Myanmar (32.7%) |

*symptomatic of mental health disorders, not clinical diagnoses

**Table 6. Grey literature findings on mainly trafficked/forced labour commercial fishers from GMS countries (n=13)**

| Author (year) | Main findings |
|---|---|
| Robertson/IOM (2011) [36] | • Expected to work 18-20 hours/day, 7 days/week. Fishing boats going to foreign waters associated with greater incidence of trafficking<br>• No toilets on small-medium boats, poor hygiene and nutrition due to need to conserve fresh water/food on long trips<br>• Physical and mental abuse common. Injuries and sickness common, but little/no medicine available, poor access to healthcare |
| Brennan/Solidarity Centre (2009) [30] | • Coastal boats: 13-14 hours at sea/day, some rest. Long-haul boats: 45-60 days at sea, working 18-24 hours/day; 36% worked 24 hours/day<br>• Witnessed physical violence by superiors: 33%; Experienced physical violence: 50%. Long-haul fishermen who were regularly beaten considered suicide<br>• Long-haul fishermen seeing fellow workers become sick/die: 36%. Not enough medicine on board, men experience "sea malaria" with fevers |
| UNIAP (2009) [46] | • Long-haul boats at sea for 2+ years. Cyclical stops made in Malaysia; Up to 3 days no rest/sleep, nutritional deprivation; Hazardous, often life-threatening working conditions; Physical violence, e.g., beatings to head/body, threats to life: 100%<br>• Deported: 100%. None screened/identified as trafficked by authorities. Some re-trafficked to palm oil/rubber plantations, some held for ransom by agents |
| Pearson/ILO (2006) [45] | • Worked 12+ hours/day: 62%. Could not refuse to do overtime: 100%. Just over 50% had regular days off<br>• When sick, 50% turned to each other for help, 33% turned to health support workers, 33% to relatives<br>• Employers unsure workers had right to leave worksite without permission: 33%; Employers believed acceptable to lock migrants in accommodation: 50%<br>• Physical violence: 14%; Verbal abuse (scolding, swearing, threats): 80%; Forced to work in fishing: 20%; Previously forced labour: 25% |
| Fujita (2010)* [56] | • Trip duration: 30-40 days, 3-5 days rest on land between trips. Long-haul boats in Indian Ocean/Indonesia for 6 months/trip, feeder boats exchanged fish for supplies once/month. N=17-18 workers per boat. Meals provided by boat owner 2-4 times/day (rice, fish), vegetables consumed every 15 days |
| ILO/ARCM (2013) [14] | • Worked 17-24 hours/day: Long-haul (28.3%), Short-haul (25.3%); Worked indefinite hours/day: Long-haul (46.2%), Short-haul (40.0%); Less than 5 hours rest in 24 hours: Long-haul (28.3%), Short-haul (38.4%). Overall, 26.4% had inadequate rest<br>• Aware of safety risks in fishing: All (91.9%), Long-haul (88.7%), Short-haul (92.7%); Ever injured: All (20.6%), Long-haul (26.4%), Short-haul (19.4%)<br>• Severely beaten: All (10.5%), Thai (8.2%), Cambodian (2.5%), Myanmar (16.3%), Long-haul (17.0%), Short-haul (8.6%)<br>• Deceived/coerced into fishing: Long-haul (16.0%), Short-haul (3.1%); Sold/transferred to another boat against will: Long-haul (5.7%), Short-haul (3.7%); Tried to escape: Long-haul (17.0%) Short-haul (9.8%); Forced labour: Thai (0%), Cambodian (9.1%), Myanmar (25.8%), Long-haul (24.5%), Short-haul (15.3%) |
| Baker/UNACT (2015) [39] | • Safe working conditions: 63.2% (lowest/sectors); Fair/good working conditions: 57.6% (2nd lowest/sectors) Fair/good Bosses: 68.0% (2nd lowest/sectors)<br>• Fair/good freedom of movement: 72.0; No restrictions on freedom of movement: 72.8; Free to quit employment: 61.6% (lowest/sectors)<br>• Violence was a problem: 26.3% (highest/sectors); Exploitative conditions: 28.8%; Cheated and/or deceived about working conditions: 44.8%; Trafficked: 21.6% |
| Verite (2015) [34] | • Worked 16 hours/day at sea or verbal/physical abuse/docked pay. Salary withheld for up to 10-19 months. 10USD/day mean salary among junior fishermen<br>• Chronic sleep deprivation, no control over rest/sleep, superiors required continuous work. Men pulled overboard by heavy nets/not recovered, especially at night<br>• Injuries and violence uncommon, usually when men are drunk/overworked, prone to lose temper/fight sometimes caused deaths at sea. Body thrown overboard to avoid contamination (captain) |
| Yea (2014) [32] | • Worked 18-22 hours/day, 7 days/week. No rest days/overtime pay for hours worked beyond 8-9 hours/day agreed in contracts. No rest days for sickness/injury.<br>• Forced to work in heavy storms/cold rooms/polar regions with no protective gear. No life jackets, life buoys locked up. Inadequate water, rotten/expired food<br>• Verbal and physical abuse, e.g., beatings (kicks, punches, slaps) to face and body by superiors |

| Author (year) | Main findings |
|---|---|
| | • Injuries via fishing hooks to face, arms and neck. Wounds sewn with needle and thread-no antiseptic/pain relief administered. Expired medicine given. Seriously injured men (cuts, lost limbs) forced to wait until vessel docked before receiving medical care. Deaths at sea among those who cannot wait/seriously ill |
| Day/HAGAR (2015) [37] | • Fishermen spent months/years at sea due to Transshipment, no freedom of movement. Tortured/electrocuted/sold to other boats for attempting escape<br>• Sickness linked to unsanitary living conditions, eating mainly raw food (inadequate cooked food)<br>• Injuries: stabs on limbs, unhealed broken bones, hooks lodged in skin. Paracetamol and basic medicines only given<br>• Poor mental health including memory loss, aggression, substance abuse linked to abuses; Few fishermen receive ongoing health assistance upon return<br>• Guilt/shame experienced for being “failed migrant”/no income, abuses witnessed or suffered, for falling victim to deception/being unable to protect self |
| EJF (2013) [52] | • Forced to work up to 20 hours/day, no wages received<br>• Threatened with weapons, physical violence by senior crew/agents (e.g. beatings, cut with knife). Witnessed torture, multiple murders |
| Stringer (2016)[h] [38] | • Excessive working hours, including 18-30 hours without breaks/rest: 76%+; Wages withheld/not paid: 51-75%<br>• Hazardous work, very bad living conditions: 76%+; Inadequate water, food (forced to eat fish bait to survive)<br>• Experienced violence/threats of violence for work mistakes/tiredness: 76%+; Sexual abuse (indecent exposure, groping, rape by superiors) common<br>• Injured crew forced to remain below deck when vessels docked, requests to see doctor denied. Lack of medical care for injured/sick men |
| Surtees (2014) [33] | • Worked min. 18 hours/day, half worked 20+hours/day, everyday. Transshipment: men kept at sea for up to 3 years<br>• Occupational hazards: falling overboard during storms due to dizziness, some deaths. Seasickness, wounds/cuts to hands from hooks, unguarded machinery. No protective equipment/gear provided for working in cold store/on deck (e.g. clothes, goggles, safety harnesses), no life jackets/buoys<br>• Forced to work when sick/injured/exhausted, beaten if caught resting/not working hard enough<br>• Dirty, unhygienic, cramped sleeping/living conditions. Inadequate/unsanitary food (forced to eat fish bait), inadequate drinking water, only seawater for bathing which caused skin conditions. Language barriers: men couldn’t express health problems/discuss medicine given by superiors<br>• Physical violence (e.g. beatings, attacked with weapons): 93.5%. Cambodians beaten more than other nationalities<br>• Psychological abuse (e.g. threatened with violence/murder, insulted/shouted at): 100%<br>• Chronic headaches, pain from broken bones (incorrectly healed), malnourishment, skin infections, lung conditions upon return<br>• Anger, fear, anxiety upon return; stress and mental health problems due to trauma experienced (physical violence, threats) or witnessed (beatings, murders). Isolation at sea/long delays in return compounds mental health problems<br>• Little/no payment received. Some men don’t return due to shame of returning with no money, transfer to other boats in hopes of earning income. Pity from community can translate to feeling supported/loved or can engender shame among returned men |

h. peer-reviewed non-health paper

*this study does not include trafficked/forced labour in the sample
