# Supplementary material for: Occupational, physical, sexual and mental health and violence among migrant and trafficked commercial fishers and seafarers from the Greater Mekong Subregion (GMS): systematic review
Source: Glob Health Res Policy. 2018 Oct 1;3:28. doi: 10.1186/s41256-018-0083-x (PMC6166293; doi:10.1186/s41256-018-0083-x)
Supplement: Supplementary file 5 — Quality Appraisal Tools. National Heart Lung & Brain Institute (NHLBI) Quality Assessment Tool for Observational Cohort and Cross-­-Sectional Studies. (PDF 65 kb) [file 41256_2018_83_MOESM5_ESM.pdf]

Quality Appraisal Tools

National Heart Lung & Brain Institute (NHLBI) Quality Assessment Tool for Observational Cohort and Cross-Sectional Studies

	Yes	No	Other*
1. Was the research question or objective clearly stated?			
2. Was the study population clearly specified and defined?			
3. Was the participation rate of eligible persons at least 50%?			
4. Were all the participants selected or recruited from the same/similar population (including the same time period)? Were inclusion and exclusion criteria for being in the study prespecified, and applied uniformly to all participants?			
5. Was a sample size justification, power description, or variance and effect estimates provided?			
6. For the analyses in this paper, were the exposure(s) of interest measured prior to the outcome(s) being measured?			
7. Was the timeframe sufficient so that one could reasonably expect to see an association between exposure and outcome if it existed?			
8. For exposures that can vary in amount or level, did the study examine different levels of the exposure as related to the outcome (e.g. categories of the exposure, or exposure measured as a continuous outcome)?			
9. Were the exposure measures (independent variables) clearly defined, valid, reliable, and implemented consistently across all study participants?			
10. Was the exposure(s) assessed more than once over time?			
11. Were the outcome measures (dependent variables) clearly defined, valid, reliable, and implemented consistently across all study participants?			
12. Were the outcome assessors blinded to the exposure status of participants?			
13. Was loss to follow up after baseline 20% or less?			
14. Were key potential confounding variables measured and adjusted statistically for their impact on the relationship between exposure(s) and outcome(s)?			
	Good	Fair	Poor
15. Given your answers to questions 1-14, what is the overall quality rating of this study in your opinion?			
16. What are the main limitations of this study in your opinion?			

*Cannot tell (not enough information given), Not reported, Not applicable

Critical Appraisal Skills Programme (CASP) Quality Appraisal tool for Qualitative Studies

	Yes	No	Cannot tell
1. Was there a clear statement of the research aim(s) of the study?			
2.a. Was a qualitative methodology appropriate?			
2.b. Was the answer to both of the above questions "No"?	End survey, mark as “Poor”	Continue Q.3	
3. Was the research design appropriate to address the research aim(s) of the study?			
4. Was the recruitment strategy appropriate to the aims of the research?			
5. Was the data collected in a way that addressed the research issue?			
6. Was the relationship between researcher and participants adequately considered?			
7. Were ethical issues taken into consideration?			
8. Was the data analysis sufficiently rigorous?			
9. Does the study have a clear statement of the findings?			
10. Does the study make clear what the value of the research is?			
	Good	Fair	Poor
11. Given your answers to questions 1-10, what is the overall quality rating of this study in your opinion?			
12. What are the main limitations of this study in your opinion?			
